# Hydrothermal treatment of yeast cell wall generates potent anti-proliferative agents targeting MCF7 breast cancer cells effectively even under culture conditions separated by a plastic wall

**DOI:** 10.1371/journal.pone.0313379

**Published:** 2025-02-14

**Authors:** Takanori Kitagawa

**Affiliations:** Agri Division and R&D Department, ASAHI BIOCYCLE CO.,LTD, Shibuya-ku, Tokyo, Japan; National Chung Cheng University, Taiwan & Australian Center for Sustainable Development Research and Innovation (ACSDRI), AUSTRALIA

## Abstract

Traditionally, the yeast cell wall (YCW) has limited applications because of its low solubility. To overcome this, a novel method was developed using a hydrothermal reaction to enhance its solubility and decrease its viscosity; this resulted in the production of a soluble form of YCW, known as the YCW treated with hydrothermal reaction (YCW-H), with broader chemical composition. However, the biological impact of YCW-H is unclear, excluding its reported plant growth-promotion by effectively regulating soil microspheres. This study investigated the potential of YCW-H to inhibit MCF-7 breast cancer cell proliferation. YCW-H demonstrated significant anti-proliferative effects on MCF7 cells, reducing cell growth by 58.7% ± 6.9 even when physically separated from the cells by a plastic wall. The observation suggests the presence of a diffusible factor against cell proliferation in YCW-H, a phenomenon not observed in the presence of untreated YCW. Reactive carbon species (RCS) generated during the hydrothermal treatment of YCW could be responsible for the effect. The addition of Fe(III) ions into YCW-H further amplified RCS production and elevated its inhibitory activity by about 10% across the plastic barrier. Radical adduct concentration of H_2_O in a tube which was incubated in YCW-H was 0.47 μmol/L, indicating that radicals migrated into the water through the plastic wall. The concentration of radical adducts in H_2_O in a tube exposed to YCW-H with Fe(III) ions further increased to 0.51 μmol/L, indicating that the growth inhibition was correlated with the increased RCS levels. Furthermore, flow cytometry analysis revealed the cytotoxic effects of YCW-H, indicating YCW-H is applicable to cancer therapy. Therefore, the findings highlight the pivotal role of RCS in the YCW-H anti-cancer activity, suggesting its potential as a promising candidate for the development of novel medical devices for cancer treatment.

## Introduction

Beer, a globally cherished beverage, is produced at a staggering rate of approximately 180 billion liters worldwide per year [1]. A crucial step in beer production is fermentation, a process catalyzed by yeast species such as *Saccharomyces cerevisiae* and *Saccharomyces pastorianus*. The yeast cells used in beer production are discarded at the end of each process, resulting in the generation of a substantial amount of yeast cell residue as byproduct [2–4]. Recycling of such byproducts may present a sustainable solution to environmental challenges.

The recycling of beer yeast cell residue is imperative for environmental stewardship and offers substantial opportunities as a versatile feedstock for a wide array of industries. Considering its rich nutrient profile; proteins, minerals, vitamins, and polysaccharides [5], beer yeast cell residue can be repurposed as a feed ingredient, a biostimulant in agriculture, or a substrate for biofuel production. The residue can be separated into two primary byproducts: yeast extract and cell wall. While yeast extract is used widely as a nutrient source, surplus amounts of yeast cell wall (YCW) remain underutilized. YCW, characterized by its poor water solubility, finds its primary application in animal feed. Its limited processability poses significant challenges for its biological activity assessment, thereby restricting its potential use in recycling. Therefore, the development of methods to overcome such challenges is necessary.

To overcome this limitation, we employed initially an enzyme treatment of YCW. The treatment improves solubility of YCW slightly, and the resulting cocktail is able to enhance plant growth by stimulating plant defense responses [6]. However, this method still falls short of enabling in-depth investigations of biological activity using mammalian cell lines. To further increase its solubility and reduce its viscosity, we developed a novel hydrothermal reaction method. The resulting soluble form of YCW, termed YCW-H, demonstrated excellent plant fertilizer properties by regulating the soil’s bacterial biosphere through a decrease in oxidation-reduction potential [7]. These findings indicated that the solubilization process generates derivatives exhibiting distinct and previously unknown biological properties. This implies that YCW-H possesses a broader spectrum of chemical components and properties compared to untreated YCW. Consequently, YCW-H enables effective *in vitro* investigations of its biological effects and potential applications.

YCW is emerging as a promising source of valuable polysaccharide components for various biomedical applications [8–11]. The major components of YCW include mannan oligosaccharides, β-glucans, and mannoproteins, which are non-filamentous glycoproteins [12–14]. Polysaccharides, in general, exhibit a wide range of biological activities, encompassing antitumor, antioxidant, immunomodulatory, anti-inflammatory, and hypoglycemic effects [15–24]. Water-soluble polysaccharide fractions derived from mushroom mycelia have demonstrated antitumor effects by inhibiting cancer cell growth [22,25,26]. Notably, polysaccharides of YCW have shown potential anticancer effects *in vivo* and possess antioxidant properties with potential applications antioxidant, antimutagen, and antigenotoxic agents [27,28]. However, a comprehensive understanding of the impacts and precise mechanisms underlying YCW biological activities remains elusive.

The biological impact of YCW-H has recently been elucidated, demonstrating its ability to modulate prokaryotic cell growth [6]. Building on such findings, the author hypothesized that YCW-H could also regulate eukaryotic cell proliferation. Here, the author reports that YCW-H inhibited MCF7 cell growth, even when physically separated from the cell culture well by a polystyrene (PS) plastic wall. Notably, the strength of growth inhibition was correlated positively with the level of reactive carbon species (RCS) in YCW-H, suggesting that RCS is a key mediator of YCW-H-induced growth inhibition in MCF7 cells through PS wall. The novel finding on the effect of RCS could have major implications for the development of innovative medical devices for cancer treatment.

## Results

### MCF7 cell growth was inhibited when cultured in a vessel adjacent to a well containing YCW-H

**Fig 1:**
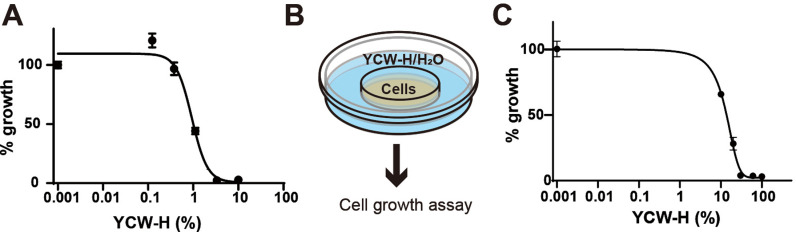
YCW-H suppressed cell growth in neighboring wells. All growth assays were performed with MCF7 cells. **(A)** Cell growth treatments with various doses (0 and 10% to 0.123% with 3 fold dilution) of YCW-H were analyzed by curve fitting. Percentage (%) growth was calculated based on the value of the wells without YCW-H as 100%. Error bars are SD (n = 4). **(B)** Schematic diagram of the assay; MCF7 cells were seeded in 3.5 cm dishes which located in 5 cm dish with various concentrations of YCW-H. **(C)** MCF7 cell growth in 0, 10, 20, 30, 60, and 100% treatments of YCW-H in adjacent treatments without lid were analyzed cell growth using curve fitting. The growth assays were performed using Crystal Violet assay. Percentage (%) growth was calculated based on the value of PBS (0%) as 100%. Error bars are SD (n = 3).

MCF7 breast cancer cells were treated with varying YCW-H concentrations of 0–10%, to assess the growth-inhibitory effect of YCW-H. EC50 is the half maximal effective concentration, which represents the concentration of a drug or other substance that induces a 50% maximal response in a biological assay. EC50 is an indicator used to compare effects of a compound in different conditions. Thus, to quantify the efficacy of the YCW-H, the EC50 for cell proliferation inhibition was measured. YCW-H inhibited MCF7 cell growth in a dose-dependent manner, with an EC50 of 0.93% (Fig 1A), indicating potent anti-proliferative activity.

Cell growth assays revealed that wells lacking YCW-H but positioned adjacent to wells with high YCW-H concentrations exhibited reduced cell growth. To confirm this reduction, growth assays were performed using a two-layer culture system consisting of an inner dish placed in an outer dish (Fig 1B). Varying concentrations of YCW-H were added to the outer dish. The cells were then cultured in the inner dish. A dose-dependent decrease in cell growth was observed, with an EC50 of 11.9%, although the sensitivity was lower than that of direct treatment with YCW-H (Fig 1C). The findings suggest that the airborne transmission of YCW-H inhibits MCF7 cell growth.

### Hydrothermal treatment conferred YCW with a growth-inhibitory effect on MCF7 cells

**Fig 2:**
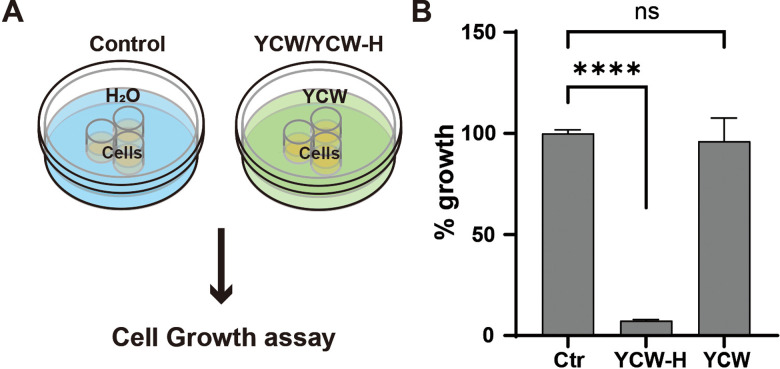
YCW-H acquired growth inhibition ability following hydrothermal reaction. **(A)** Schematic diagram of the assay; YCW-H/YCW or H_2_O (Control) were filled in a dish outside of triple wells. **(B)** Percentage (%) growth over a 3-day culture was calculated based on the value of control (Ctr) as 100%. Error bar is SD (n = 3). p-value was indicated by ****: < 0.0001 and ns: non-significance. Statistical significance was evaluated using a one-way ANOVA test and the Brown-Forsythe test in GraphPad Prism.

The growth inhibitory effects of YCW-H and YCW were compared with those of H₂O (control, 100%) to determine whether hydrothermal treatment was required. YCW was sterilized via autoclaving. MCF7 cells were seeded into three wells of a triple-well dish. After one day, the space around the culture wells was filled with H₂O, YCW-H, or YCW (Fig 2A). Cell proliferation was inhibited significantly to 7.4% ± 0.32 in the presence of YCW-H compared to that of the control, whereas cells treated with YCW exhibited no significant inhibition (96.2% ± 8.1) (Fig 2B). Therefore, the hydrothermal treatment of YCW endows it with the ability to inhibit cell growth.

### YCW-H can inhibit MCF7 cell growth by passing through plastic walls

**Fig 3:**
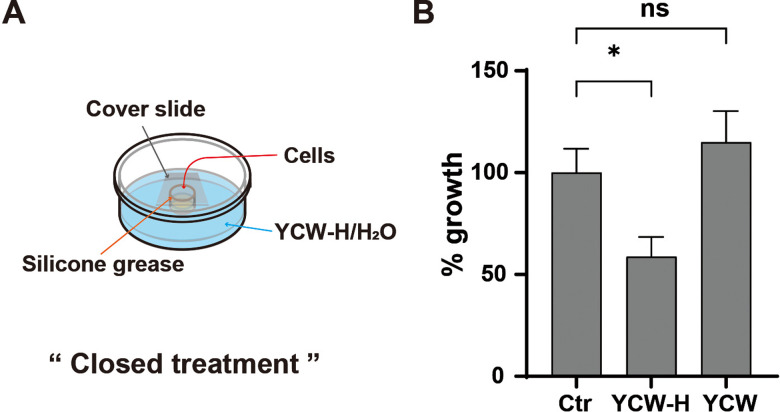
YCW-H suppressed cell growth from neighbor wells. **(A)** Schematic diagram of the assay; YCW-H/YCW or H_2_O (Control) were filled in a dish outside of a well. The well was closed (Close) using a cover slide with silicon grease. **(B)** Percentage (%) growth for a 3 day culture was calculated based on the value of control (Ctr) as 100%. Error bar is SD (n = 3). p-value was indicated as * : P = 0.016 and ns: non-significance: P = 0.34. Statistical significance was evaluated using a one-way ANOVA test and the Brown-Forsythe test in GraphPad Prism.

To further investigate whether the growth inhibition by YCW-H occurred via airborne transmission, a growth assay was performed under completely isolated conditions. MCF7 cells were seeded in a single-well dish, and the culture well was sealed with a coverslip using silicone grease to prevent air movement. The space outside the well was then filled with H₂O (control), YCW, or YCW-H (Fig 3A). The treatment from an outside well with a coverslip was termed “closed treatment.” YCW-H significantly inhibited MCF7 cell growth to 58.7% ± 6.9 under closed treatment, compared with that observed in the control, whereas YCW had no effect on MCF7 cell growth (115.0% ± 10.8) (Fig 3B). Thus, YCW-H inhibited cell growth even when the MCF7 cells were completely segregated from YCW-H.

### YCW-H contains reactive carbon species (RCS), whose generation is regulated by iron ions

**Fig 4:**
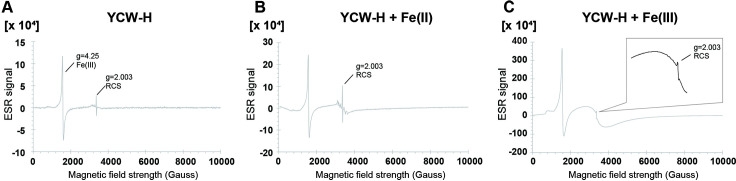
YCW-H possessed reactive carbon species. **(A)** YCW-H, **(B)** YCW-H and Fe (II) (46.3 mM) and **(C)** YCW-H and Fe (III) (46.3 mM) were analyzed by electrospin resonance (ESR) spectroscopy. Magnified image of the RCS peak is shown in the box. X-axis indicates magnetic field strength (Gauss). Y-axis represents units of the ESR signal. Value of a constant of proportionality, g, is the property of the electron.

The author hypothesizes that YCW-H contains radical species that similarly influence cell proliferation, since studies have shown that reactive nitrogen species (RNS) prevent cell death through airborne transmission [29]. Therefore, electron spin resonance (ESR) spectroscopy was used to investigate the presence of radical species in YCW-H. The electronic g-factor, a key identifier for radical species, indicated that YCW-H contains RCS and Fe(III) complex, with g-factors of 2.003 and 4.25, respectively (Fig 4A) [30–32]. The next step was to identify the inducer of RCS production in YCW-H. The influence of iron (Fe) ions on RCS production was investigated, since Fe ions can generate radicals, including RCS, in certain conditions [33–36]. The addition of FeSO_4_, Fe(II), to YCW-H increased the RCS levels to 2.7 μmol/L compared with 1.9 μmol/L that was observed in the presence of YCW-H alone (Fig 4B, Table 1). Adding Fe_2_(SO_4_)_3_, “Fe(III),” into YCW-H increased the RCS level to 8.5 μmol/L, and a broad signal from hydrated Fe(III) was observed (Fig 4C, Table 1). The concentration of Fe(III) complex increased from 0.15 to 0.29 mmol/L upon addition of Fe(II), suggesting that Fe(II) oxidation increases Fe(III) levels. The findings suggest that Fe(II) and Fe(III) contribute to RCS production in the YCW-H.

**Table 1:** Concentration of RCS and Fe(III) complex measured by ESR analysis.

Samples	RCS: g = 2.003 (μmol/L)	Fe(III) complex: g = 4.25 (mmol/L)
YCW-H	1.9	0.15
YCW-H + Fe(II)	2.7	0.29
YCW-H +Fe(III)	8.5	11

The substantial change of about 10%, given the instrument’s precision of ± 5%, was deemed an effective difference.

### RCS can migrate through plastic walls during incubation

To investigate the migration of radical species to neighboring wells under closed conditions, H₂O containing the spin trap N-tert-butyl-α-phenylnitrone in a polystyrene tube which was immersed in YCW-H was analyzed by ESR. The ESR analysis revealed an increase in the concentration of radical adducts from 0.43 (in H_2_O) to 0.47 μmol/L (in YCW-H). The addition of Fe₂(SO_4_)₃ (46.3 mM) to YCW-H further increased the adduct concentration to 0.51 μmol/L (Table 2). The findings suggest that radical species can permeate polystyrene walls, demonstrating their ability to migrate between completely separate compartments.

**Table 2:** Concentration of radical adducts with PBN measured by ESR analysis.

Samples	Radical adducts (μmol/L)
H_2_O	0.43
YCW-H	0.47
YCW-H + Fe(III)	0.51

The substantial change of about 10%, given the instrument’s precision of ± 5%, was deemed an effective difference.

### Ferrous and ferric ions accelerated MCF7 growth inhibition in YCW-H adjacent treatment under open and closed conditions

**Fig 5:**
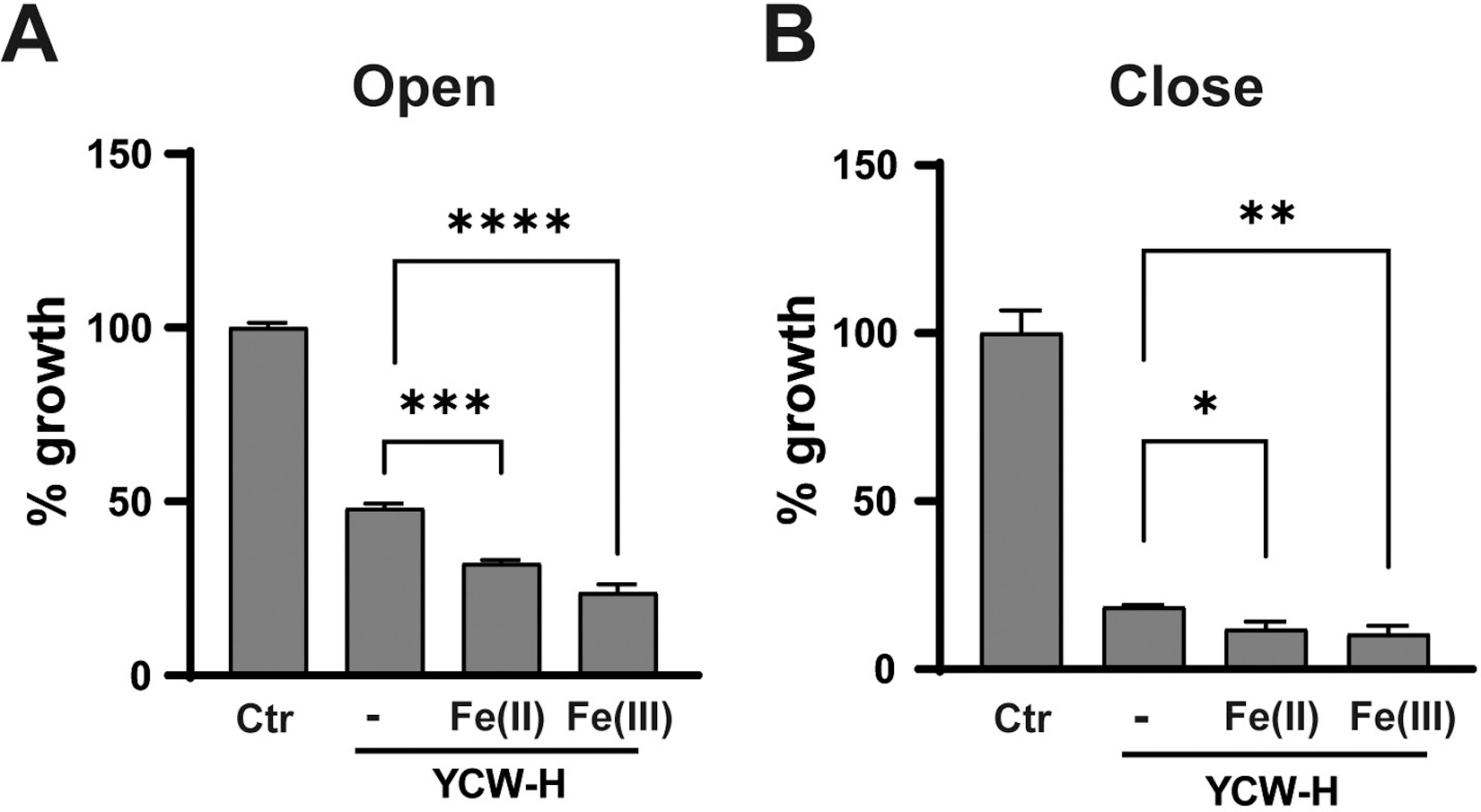
Fe(II) and Fe(III) were required for YCW-H growth inhibition. **(A)** MCF7 cells were seeded in the wells of well dishes under open treatments. The outer space was filled with H_2_O (Ctr), and H_2_O (-), 40 mM of Fe(II) or Fe(III) in 25% YCW-H for 3 days. H_2_O alone was used as a control (100%). P-value is represented as ****: P < 0.0001, ***: P = 0.0005. **(B)** MCF7 cells were seeded in the wells of well dishes under close treatments. The outer space was filled with H2O (Ctr), and H_2_O (-), 40 mM Fe(II), and 40 mM Fe(III) in YCW-H. Growth assays were performed using a Crystal Violet assay. Percentage (%) growth was calculated based on the value of H_2_O as 100%. Error bar is SD (n = 3). P-value is represented as**: P = 0.005 ** and * : P = 0.01). Statistical significance was evaluated using one-way ANOVA test and the Brown-Forsythe test in GraphPad Prism.

The author hypothesized that the RCS in YCW-H could influence its inhibitory activity through plastic walls. Because Fe(II) and Fe(III) can increase the level of RCS, the effects of adding these ions on the growth-suppressive activity of YCW-H was investigated. Triple-well dishes were used for growth assays in the open treatment (Fig 5A). YCW-H (25%) treatment alone inhibited growth to 48% ± 1.4 of the control; however, addition of Fe(II) to YCW-H reduced the cell growth to 32% ± 1.1, while the addition of Fe(III) reduced it to 24% ± 2.3. This suggests that adding Fe(II) or Fe(III) to YCW-H accelerates the inhibition of cell proliferation compared to that of YCW-H alone under the open treatment conditions (Fig 5A).

To further validate the effects of RCS via the combination of Fe(II) and Fe(III), the effects of the addition of Fe(II) and Fe(III) to YCW-H on MCF7 cell growth were explored. YCW-H alone inhibited proliferation by 18.6% ± 0.46, YCW-H with Fe(II) by 11.9% ± 1.5, and YCW-H with Fe(III) by 10.4% ± 1.8 compared with that of the control. This indicates that the addition of Fe(II) or Fe(III) enhances the growth-inhibitory ability of YCW-H against MCF7 cells under close treatment conditions (Fig 5B). Additionally, the increase in RCS levels owing to the presence of Fe(II) and Fe(III) correlated with the extent of growth inhibition (Table 1). Thus, RCS activated by iron ions may play a key role in inhibiting MCF7 cell growth, even under conditions of complete separation.

### YCW-H was cytotoxic to MCF7 cells under open and closed YCW-H adjacent treatment conditions

**Fig 6:**
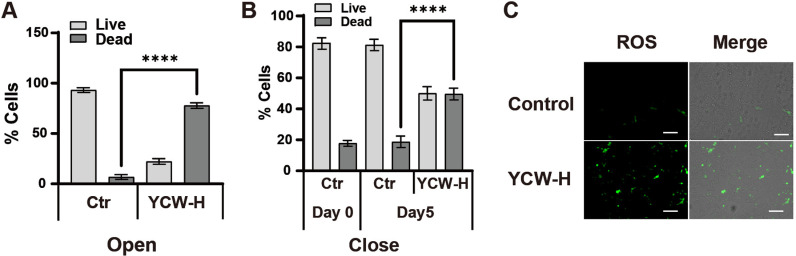
Adjacent YCW-H treatment induced cell death and ROS. **(A)** Number of live and dead cells with and without YCW-H were measured using Guava® with ViaCount™. The cells were cultured for 4 days under Open condition. Error bar is SD (n = 3). P-value is represented as ****: p < 0.0001. **(B)** Number of live and dead cells with and without YCW-H after 5 days in Close condition. Day 0 is one day before the treatment was started. Error bar is SD (n = 3). P-value is indicated as ****; p < 0.0001. Statistical significance was evaluated using a one-way ANOVA test and the Brown-Forsythe test in GraphPad Prism. **(C)** ROS induction was detected using an ROS detection kit. MCF7 cells were cultured with and without YCW-H treatment for 3 days in close condition. Green fluorescence indicated ROS induction in cells (ROS). Images of brightfield and fluorescence were overlaid (Overlay). Representative images from triplicate assays are indicated. The white bar indicates 100 μm.

To determine whether the growth suppression via YCW-H adjacent treatment was due to cytotoxicity, the percentages of live and dead cells in the treated cells were analyzed using flow cytometry with the detection reagent ViaCount^TM^. In open conditions, the presence of 25% YCW-H in the surrounding space resulted in 77.8% ± 2.3 of MCF7 cell death, compared to 6.8% ± 1.9 in the control group treated with H₂O (Fig 6A). Under the “closed treatment”, 49.6% ± 3.1 of the treated cells underwent cell death, compared to 18.7% ± 3.0 of the control cells (Fig 6B). Furthermore, at the exponential phase (Day 0), the percentage of dead cells was 18% ± 1.5, similar to the percentage of untreated cells, suggesting that the closed treatment did not affect MCF7 cell proliferation (Fig 6B). Reactive oxygen species (ROS) levels in the YCW-H adjacent treatment were examined under closed treatment conditions, since ROS activation induces cell death [34]. ROS were elevated in cells subjected to YCW-H closed treatment before cell death induction but not in untreated cells (Fig 6C). The increase in ROS levels was correlated with the number of dead cells, suggesting that ROS activation could be involved in RCS-mediated cell death. Therefore, the growth inhibition observed in the YCW-H adjacent treatments was deemed to be the result of cytotoxicity.

## Discussion

The present study investigated the effects of YCW-H derived from beer yeast remains on the cancer cell growth and revealed that YCW-H had an active ingredient RCS which was worked as an anti-cancer agent. Interestingly, a raw material of beer, Hops has been used as a medicinal plant in traditional medicine for anxiety, insomnia, mild pain and dyspepsia treatment [37]. YCW-H might have some agents from the beer raw material and contain a wider variety of agents than those of the yeast cell wall. Therefore, YCW subjected to hydrothermal treatment has unique chemical contents and properties, such as RCS, with MCF7 cell growth inhibition.

The results indicate that YCW-H induces cytotoxicity in cancer cells regardless of their physical proximity, including in completely isolated culture wells. RCS generated by YCW-H would be a key mediator of the growth-inhibitory effects. Whereas carbon radicals are implicated in diverse biochemical reactions, their biological function remains poorly understood [38–42]. Notably, they are essential for initiating endoperoxide-induced apoptosis [43]. Cytochrome P450 reductase, a key enzyme involved in xenobiotic metabolism, regulates ferroptotic cell death by producing lipid-derived RCS, including alkyl radicals, which function as dynamic signaling molecules [40,41,43–47]. Therefore, RCS generated by YCW-H could react with cellular intermediates, acting as signaling molecules to trigger cell death.

The presence of Fe(III) enhanced the RCS-generation activity of YCW-H; the induction of RCS generation proportionally enhanced the growth-inhibitory effects of YCW-H under open and closed conditions. Adding Fe(II) significantly promotes the growth inhibitory effects of MCF7 cells in addition to increasing the amounts of Fe(III) complexes and RCS. The effects of Fe (II) addition may be attributed to the elevated Fe(III) formation. Such elevation of Fe(III) results from a redox reaction between Fe(II) and Fe(III), which can be exploited for growth inhibition. Fe(II) suspensions, when exposed to O_2_ under certain conditions, undergo spontaneous oxidation at room temperature [48]. In contrast, the reduction of Fe(III) to Fe(II) occurs with extremely low efficiency owing to its minimal redox potential [48]. Thus, Fe(II) supplementation in YCW-H catalyzes the conversion to Fe(III), enhancing YCW-H-dependent cell death via inducing RCS. The findings support the unique growth inhibitory effect of RCS and suggest that Fe(III) enhances the YCW-H activity.

ESR analysis revealed that YCW-H contains ferric ions as Fe(III) complexes. USDA data shows that barley malt has 4.7 mg of iron per 100 g, suggesting that barley malt is a potential iron-source in YCW-H. Furthermore, *S. cerevisiae* uses mannoproteins present in the cell wall to facilitate the uptake of Fe(III) complexes, thereby accumulating more Fe(III) in the cell wall [49,50]. Therefore, the cell walls of the yeast may retain a sufficient amount of Fe(III) and organic carbon after fermentation, forming Fe(III) complexes.

A YCW-H treatment in adjacent wells increases intracellular ROS levels, leading to cell death, while the factors responsible for activating ROS and cell death remain unknown. Cellular ROS are endogenously produced during mitochondrial oxidative phosphorylation in response to various biological processes, such as xenobiotic metabolism, phagocytosis, and arginine metabolism [51–53]. Excessive cellular ROS can damage key cellular components, including proteins, nucleic acids, and organelles, which may ultimately trigger cell death [54]. Both intrinsic and extrinsic pathways converge on ROS-mediated damage, making ROS a critical factor in cancer cell death [55]. Peroxyl radicals, formed by the direct reaction of oxygen with alkyl radicals in L-carbon-centered lipid radicals, are implicated in ferroptosis via lipid ROS controlled by singlet oxygen, supporting our finding [40]. The present study shows that cell growth inhibition by YCW-H is positively correlated with an increase in RCS levels. Therefore, RCS-containing compounds in the YCW-H adjacent treatments could induce intracellular ROS production, leading to cell death.

Our findings indicate that the RCS-containing factors of YCW-H may migrate through the plastic walls of polystyrene culture plates. In this case, chemical transport in the polystyrene wall would be necessary. The migration requires three steps: absorption into the polystyrene, diffusion through the material, and finally, release into the adjacent well. Chemicals are reportedly absorbed into polystyrene assay plates, since this chemical adsorption to polystyrene is often responsible for most of the reduction in the levels of the observed chemicals in medium in *in vitro* assays by uneven influx and efflux rates within the same well [56–60]. For the transmigration to a new well, chemical diffusion in polystyrene would occur along a chemical gradient between the two wells driven by chemical concentrations in each medium. The chemicals would eventually be released to a fresh medium, when they reach the opposite side. Alternatively, RCS would be transferred by radical chain reaction, since thermal degradation of most of the polymers is a typical radical chain mechanism [61]. Therefore, the chemicals with RCS-containing factors could be transferred across the plastic wall.

In conclusion, the RCS from YCW-H, enhanced by iron ions, exhibited potent anti-proliferative and pro-apoptotic effects via cytotoxicity. The cytotoxic activity of YCW-H RCS through the plastic barrier is a unique finding with potential cancer therapeutic application. Chemotherapy, commonly used in the treatment of various cancers, including skin and breast cancer, often has side effects such as hair loss, diarrhea, vomiting, chest pain, constipation, difficulty breathing, fatigue, mucositis, and rash. There is an urgent need for innovative approaches to the development of novel cancer therapeutics. The integration of new techniques into such efforts holds great promise for unraveling the complex nature of cancer and identifying novel therapeutic targets and modalities [62–66]. To improve patient quality of life, future investigations should focus on developing innovative therapies that minimize such side effects. The agents capable of traversing plastic walls represent a novel avenue for cell inhibition and hold promise for the development of noninvasive medical devices for cancer treatment.

## Materials and methods

### Cell culture

The breast cancer cell lines, MCF7, were purchased from the Japanese Collection of Research Bioresources Cell Bank (JCRB, Osaka Japan). MCF7 cells were cultured in DMEM supplemented with 10% fetal bovine serum and 1% penicillin-streptomycin at 37°C using 75 cm² flasks in a sterile incubator at 37°C with 5% CO_2_ under saturation humidity. When the cells reached 70%–80% confluence, they were detached with 0.25% trypsin and passaged [67]. Cancer cells in the logarithmic growth phase were obtained for the experiment. Regular testing of mycoplasma contamination was performed in the cell lines using a CycleavePCR™ Mycoplasma Detection Kit (Takara, Shiga Japan), and only mycoplasma free cells were used for experiments.

### Preparation of YCW

Yeast cell wall (ASAHI Group Foods Ltd., Tokyo Japan) were prepared into a 15% (w/v) solution with water and then sterilized by autoclaving at 121 °C for 20 min.

### Hydrothermal reaction of YCW - YCW-H

Yeast cell wall (ASAHI Group Foods Ltd., Tokyo Japan) were prepared into a 15% (w/v) solution with water. The solution was gradually heated in an industrial autoclave from room temperature to 180 °C and 1.6 MPa over 2 h. Once the target temperature and pressure were achieved, autoclaving was halted immediately, and the solution was allowed to return to room temperature.

### Cell growth assay

Cell growth was measured using a crystal violet assay. Cells were fixed with 100 μL of 1% paraformaldehyde #163-20145 (WAKO, Osaka Japan) and stained with 100 μL of 0.2% crystal violet # 15192 (MUTO PURE CHEMICALS, Tokyo Japan) after cell cultures were completed. The crystal violet was extracted by 100 μL of 50% ethanol. The absorption of the extracts was measured at 570 nm using a SpectraMax® M3 (Molecular Devices, San Jose CA USA). *Cell culture for 96 well growth assay*; MCF7 cell lines were seeded with 4,000 cells in each well in 96 well plates and incubated for one day in 100 μL of DMEM supplemented with 10%FBS, 1% penicillin and streptomycin at 37°C in 5% CO_2_. Afterward, 100 μL of DMEM with 10%FBS, 1% penicillin and streptomycin with and without YCW-H were added into the cell culture. Various percentages of YCW-H were diluted in DMEM. The cells were subsequently incubated for 3–5 days. *Cell culture with adjacent treatment under open conditions in culture dishes*; 8 × 10^4^ MCF7 cells were seeded in 3.5 cm dishes which were located in 5 cm dishes with various concentrations of YCW-H. Varying concentrations of YCW-H which were diluted in PBS —10, 20, 30, 60, and 100%—as well as PBS (0%), were added to the outer dish. *Cell culture with adjacent treatment under open and close conditions in well dishes;* MCF7 cell lines were seeded with 4,000 cells in each well (φ8 mm) on a triple well dish (φ35 mm) # 3970-103 (AGC/Iwaki, Shizuoka Japan) and incubated for one day in 100 μL of DMEM supplemented with 2%FBS, 1% penicillin and streptomycin at 37°C in 5% CO_2_. For the open culture condition, the cells were subsequently incubated with and without 2 mL of YCW-H (25%) in the outside space of triple-wells for 3–5 days. For the YCW-H adjacent treatment, “close” condition, the wells, in which cells were cultured, were sealed by silicone grease with φ13 mm cover slides and then incubated with and without YCW-H in the outside space of a well for 3–5 days. The percentage of growth is expressed relative to the untreated control (set at 100%). A value less than 100% indicates growth inhibition.

### Cell counts for live and dead cells

To harvest adherent cells, cells were detached with trypsin and mixed with the culture medium. All the cell mixtures were resuspended at 1 x 10^6^ cells/mL with culture medium. ViaCount reagent (380 μL) # SKU 4000-0040 (Cytek Biosciences, Fremont CA USA) was added to 20 μL of the cell suspension and incubated at 25°C for 10 min. Cell counting was performed automatically by Guava® easyCyte^TM^ 5HT (Merck Millipore, Burlington, MA, USA) and the analyses were provided plots separating live and dead cells by the Guava® ViaCount™ Software Module (Merck Millipore).

### ROS detection

An ROS Assay Kit -Highly Sensitive DCFH-DA # R252 (Dojindo, Kumamoto Japan) was used to detect intercellular ROS activation. MCF7 cells (5,000 cells) were seeded into triple well dishes # 3970-103 (AGC/Iwaki, Shizuoka Japan) and incubated with and without YCW-H in the outer space of triple-wells for 3 days under close conditions. After the culture medium was removed, cells were stained with DCFH-DA dye according to the manufacturer’s instructions. Fluorescence signals were imaged using 20 × lens in a BZ-X710 fluorescence microscope (Keyence, Osaka Japan).

### Rapid-freezing method for ESR analysis

YCW-H (600 μL) was frozen rapidly by squirting it through a needle (i.d. 0.7 mm) into the cooling liquid at about 20 K according to the method reported previously [68]. A series of cocktails of YCW-H mixed with H_2_O, Fe(SO_4_) (46.3 mM), or Fe_2_(SO_4_)_3_ (46.3 mM) were used for analysis. Fe(SO_4_)· 7H_2_O, purity ≥ 99% (SIGMA, St. Louis, USA) and Fe_2_(SO_4_)_3_· _n_H_2_O, purity 60.0–80.0% (mass/mass) [as Fe_2_(SO_4_)_3_] (WAKO, Osaka, Japan) were used.

### ESR measurements

The samples were analyzed using an EMXplus ESR spectrometer (Bruker, Billerica, MA USA) using an X-band standard frequency of 8.8–9.6 GHz. To identify the peaks, the signal components were analyzed by the WinEPR analysis software associated with the ESR instrument (Bruker, Billerica, MA USA). The following ESR parameters were used: a frequency of 9.42 GHz, center field of 335 ± 10 mT, modulation frequency of 100 kHz, time constant of 0.03 s, and power of 5.00 mW. Aqueous samples were loaded into an LC-12 aqueous quartz flat cell.

### Preparation of H₂O containing the spin trap N-tert-butyl-α-phenylnitrone immersed in YCW-H

YCW-H was placed in a 15-mL polystyrene tube and immersed in a 5-mL polystyrene tube containing 500 μL of H₂O with 2 μM of the spin trap N-tert-butyl-α-phenylnitrone for 3 days. Purity of N-tert-butyl-α-phenylnitrone (TIC, Tokyo, Japan) was > 98.0%(T)(HPLC).

## Supporting information

S1 Data ESR Data for Table 2.
(XLSX)

S2 Data ESR Fig 4, Table 1 data. (XLSX)

S3 Data Crystal violet assay. (XLSX)

S4 Data Experiments (Crystal violet assay). (XLSX)

S5 Data Experiments (Crystal violet assay). (XLSX)

S6 Data Experiments (Crystal violet assay). (XLSX)

S7 Data Experiments (Crystal violet assay). (XLSX)

S8 Data Live/Dead cells 4 days incubation. (XLSX)

S9 Data FCM analysis (%). (XLSX)

S10 Data Experiments (Crystal violet assay). (XLSX)

## References

[1] Barth SJ. BarthHaas report. 2022.

[2] Dequin S, Casaregola S. The genomes of fermentative Saccharomyces. C R Biol. 2011;334(8–9):687–93. doi: 10.1016/j.crvi.2011.05.019 21819951

[3] Rainieri S, Kodama Y, Kaneko Y, Mikata K, Nakao Y, Ashikari T. Pure and mixed genetic lines of *Saccharomyces bayanus* and *Saccharomyces pastorianus* and their contribution to the lager brewing strain genome. Appl Environ Microbiol. 2006;72(6):3968–74. doi: 10.1128/AEM.02769-05 16751504 PMC1489639

[4] Tamai Y, Momma T, Yoshimoto H, Kaneko Y. Co-existence of two types of chromosome in the bottom fermenting yeast, *Saccharomyces pastorianus*. Yeast. 1998;14(10):923–33. doi: 10.1002/(SICI)1097-0061(199807)14:10<923::AID-YEA298>3.0.CO;2-I 9717238

[5] Marson GV, de Castro RJS, Belleville M-P, Hubinger MD. Spent brewer’s yeast as a source of high added value molecules: a systematic review on its characteristics, processing and potential applications. World J Microbiol Biotechnol. 2020;36(7):95. doi: 10.1007/s11274-020-02866-7 32583032

[6] Narusaka M, Minami T, Iwabuchi C, Hamasaki T, Takasaki S, Kawamura K, et al. Yeast cell wall extract induces disease resistance against bacterial and fungal pathogens in *Arabidopsis thaliana* and Brassica crop. PLoS One. 2015;10(1):e0115864. doi: 10.1371/journal.pone.0115864 25565273 PMC4286235

[7] Kitagawa T. Seibutu-kougaku Kaishi (Japanese). 2018;96:461.

[8] Bastos R, Oliveira PG, Gaspar VM, Mano JF, Coimbra MA, Coelho E. Brewer’s yeast polysaccharides - A review of their exquisite structural features and biomedical applications. Carbohydr Polym. 2022;277:118826. doi: 10.1016/j.carbpol.2021.118826 34893243

[9] Liu Y, Wu Q, Wu X, Algharib SA, Gong F, Hu J, et al. Structure, preparation, modification, and bioactivities of β-glucan and mannan from yeast cell wall: a review. Int J Biol Macromol. 2021;173:445–56. doi: 10.1016/j.ijbiomac.2021.01.125 33497691

[10] Ferreira IMPLVO, Pinho O, Vieira E, Tavarela JG. Brewer’s Saccharomyces yeast biomass: characteristics and potential applications. Trends Food Sci Technol. 2010;21(2):77–84. doi: 10.1016/j.tifs.2009.10.008

[11] Puligundla P, Mok C, Park S. Advances in the valorization of spent brewer’s yeast. Innov Food Sci Emerg Technol. 2020;62:102350. doi: 10.1016/j.ifset.2020.102350

[12] Cabib E, Roberts R, Bowers B. Synthesis of the yeast cell wall and its regulation. Annu Rev Biochem. 1982;51:763–93. doi: 10.1146/annurev.bi.51.070182.003555 7051965

[13] Cawley TN, Ballou CE. Identification of two *Saccharomyces cerevisiae* cell wall mannan chemotypes. J Bacteriol. 1972;111(3):690–5. doi: 10.1128/jb.111.3.690-695.1972 4559821 PMC251341

[14] Kollár R, Reinhold BB, Petráková E, Yeh HJ, Ashwell G, Drgonová J, et al. Architecture of the yeast cell wall. Beta(1-->6)-glucan interconnects mannoprotein, beta(1-->)3-glucan, and chitin. J Biol Chem. 1997;272(28):17762–75. doi: 10.1074/jbc.272.28.17762 9211929

[15] Chaisuwan W, Phimolsiripol Y, Chaiyaso T, Techapun C, Leksawasdi N, Jantanasakulwong K, et al. The antiviral activity of bacterial, fungal, and algal polysaccharides as bioactive ingredients: potential uses for enhancing immune systems and preventing viruses. Front Nutr. 2021;8:772033. doi: 10.3389/fnut.2021.772033 34805253 PMC8602887

[16] Guo R, Chen M, Ding Y, Yang P, Wang M, Zhang H, et al. Polysaccharides as potential anti-tumor biomacromolecules -a review. Front Nutr. 2022;9:838179. doi: 10.3389/fnut.2022.838179 35295918 PMC8919066

[17] Jiang Y, Zhou W, Zhang X, Wang Y, Yang D, Li S. Protective effect of blood cora polysaccharides on H9c2 rat heart cells injury induced by oxidative stress by activating Nrf2/HO-1 signal pathway. Front Nutr. 2021;8:632161. doi: 10.3389/fnut.2021.632161 33738296 PMC7960668

[18] Muszyńska B, Grzywacz-Kisielewska A, Kała K, Gdula-Argasińska J. Anti-inflammatory properties of edible mushrooms: a review. Food Chem. 2018;243:373–81. doi: 10.1016/j.foodchem.2017.09.149 29146352

[19] Nauts HC, Swift WE, Coley BL. The treatment of malignant tumors by bacterial toxins as developed by the late William B. Coley, M.D., reviewed in the light of modern research. Cancer Res. 1946;6:205–16. 21018724

[20] Pillemer L, Ross OA. Alterations in serum properdin levels following injection of Zymosan. Science. 1955;121(3151):732–3. doi: 10.1126/science.121.3151.732 14372981

[21] Ren L, Zhang J, Zhang T. Immunomodulatory activities of polysaccharides from Ganoderma on immune effector cells. Food Chem. 2021;340:127933. doi: 10.1016/j.foodchem.2020.127933 32882476

[22] Suzuki I, Itani T, Ohno N, Oikawa S, Sato K, Miyazaki T, et al. Effect of a polysaccharide fraction from *Grifola frondosa* on immune response in mice. J Pharmacobiodyn. 1985;8(3):217–26. doi: 10.1248/bpb1978.8.217 3891963

[23] Zhao R, Gao X, Cai Y, Shao X, Jia G, Huang Y, et al. Antitumor activity of *Portulaca oleracea* L. polysaccharides against cervical carcinoma in vitro and in vivo. Carbohydr Polym. 2013;96(2):376–83. doi: 10.1016/j.carbpol.2013.04.023 23768576

[24] Zhou W-J, Wang S, Hu Z, Zhou Z-Y, Song C-J. Angelica sinensis polysaccharides promotes apoptosis in human breast cancer cells via CREB-regulated caspase-3 activation. Biochem Biophys Res Commun. 2015;467(3):562–9. doi: 10.1016/j.bbrc.2015.09.145 26431878

[25] Cui FJ, Tao WY, Xu ZH, Guo WJ, Xu HY, Ao ZH, et al. Structural analysis of anti-tumor heteropolysaccharide GFPS1b from the cultured mycelia of *Grifola frondosa* GF9801. Bioresour Technol. 2007;98(2):395–401. doi: 10.1016/j.biortech.2005.12.015 16459075

[26] Do TTH, Lai TNB, Stephenson SL, Tran HTM. Cytotoxicity activities and chemical characteristics of exopolysaccharides and intracellular polysaccharides of *Physarum polycephalum* microplasmodia. BMC Biotechnol. 2021;21(1):28. doi: 10.1186/s12896-021-00688-5 33773573 PMC8005236

[27] Fortin O, Aguilar-Uscanga B, Vu KD, Salmieri S, Lacroix M. Cancer chemopreventive, antiproliferative, and superoxide anion scavenging properties of *Kluyveromyces marxianus* and *Saccharomyces cerevisiae* var. boulardii cell wall components. Nutr Cancer. 2018;70(1):83–96. doi: 10.1080/01635581.2018.1380204 29144773

[28] Kogan G, Pajtinka M, Babincova M, Miadokova E, Rauko P, Slamenova D, et al. Yeast cell wall polysaccharides as antioxidants and antimutagens: can they fight cancer? Neoplasma. 2008;55(5):387–93. 18665748

[29] Mizuno H, Kubota C, Takigawa Y, Shintoku R, Kannari N, Muraoka T, et al. 2,2,6,6-Tetramethylpiperidine-1-oxyl acts as a volatile inhibitor of ferroptosis and neurological injury. J Biochem. 2022;172(2):71–8. doi: 10.1093/jb/mvac044 35512114

[30] Hirota Y, Haida M, Mohtarami F, Takeda K, Iwamoto T, Shioya S, et al. Implication of ESR signals from ceruloplasmin (Cu(2+)) and transferrin (Fe(3+)) in pleural effusion of lung diseases. Pathophysiology. 2000;7(1):41–5. doi: 10.1016/s0928-4680(99)00033-4 10825684

[31] Kono Y, Kashine S, Yoneyama T, Sakamoto Y, Matsui Y, Shibata H. Iron chelation by chlorogenic acid as a natural antioxidant. Biosci Biotechnol Biochem. 1998;62(1):22–7. doi: 10.1271/bbb.62.22 9501514

[32] Tian L, Koshland CP, Yano J, Yachandra VK, Yu ITS, Lee SC, et al. Carbon-centered free radicals in particulate matter emissions from wood and coal combustion. Energy Fuels. 2009;23(5):2523–6. doi: 10.1021/ef8010096 19551161 PMC2700017

[33] Arai N, Narasaka K. Development of new methods for generation of radical species by one-electron oxidation with metallic oxidants toward construction of carbon skeletons. J Syn Org Chem Jpn. 1996;54(11):964–75. doi: 10.5059/yukigoseikyokaishi.54.964

[34] Jiang H, Lai W, Chen H. Generation of carbon radical from iron-hydride/alkene: exchange-enhanced reactivity selects the reactive Spin State. ACS Catal. 2019;9(7):6080–6. doi: 10.1021/acscatal.9b01691

[35] Klebanoff SJ, Waltersdorph AM, Michel BR, Rosen H. Oxygen-based free radical generation by ferrous ions and deferoxamine. J Biol Chem. 1989;264(33):19765–71. doi: 10.1016/s0021-9258(19)47178-0 2555330

[36] Feng Y, Wu D, Li H, Bai J, Hu Y, Liao C, et al. Activation of persulfates using siderite as a source of ferrous ions: sulfate radical production, stoichiometric efficiency, and implications. ACS Sustainable Chem Eng. 2018;6(3):3624–31. doi: 10.1021/acssuschemeng.7b03948

[37] Jiang C, Xie N, Sun T, Ma W, Zhang B, Li W. Xanthohumol inhibits TGF-β1-induced cardiac fibroblasts activation via mediating PTEN/Akt/mTOR signaling pathway. Drug Des Devel Ther. 2020;14:5431–9. doi: 10.2147/DDDT.S282206 33324040 PMC7732164

[38] Ning S, Liu Z, Chen M, Zhu D, Huang Q. Nanozyme hydrogel for enhanced alkyl radical generation and potent antitumor therapy. Nanoscale Adv. 2022;4(18):3950–6. doi: 10.1039/d2na00395c 36133353 PMC9470029

[39] Seren S, Joly J-P, Voisin P, Bouchaud V, Audran G, Marque SRA, et al. Neutrophil elastase-activatable prodrugs based on an alkoxyamine platform to deliver alkyl radicals cytotoxic to tumor cells. J Med Chem. 2022;65(13):9253–66. doi: 10.1021/acs.jmedchem.2c00455 35764297 PMC9289877

[40] Zhang X, Wu L, Zhen W, Li S, Jiang X. Generation of singlet oxygen via iron-dependent lipid peroxidation and its role in Ferroptosis. Fundam Res. 2021;2(1):66–73. doi: 10.1016/j.fmre.2021.07.008 38933913 PMC11197759

[41] Anderson RF, Yadav P, Shinde SS, Hong CR, Pullen SM, Reynisson J, et al. Radical chemistry and cytotoxicity of bioreductive 3-substituted quinoxaline Di-N-Oxides. Chem Res Toxicol. 2016;29(8):1310–24. doi: 10.1021/acs.chemrestox.6b00133 27380897

[42] Arroyo CM, Kramer JH, Leiboff RH, Mergner GW, Dickens BF, Weglicki WB. Spin trapping of oxygen and carbon-centered free radicals in ischemic canine myocardium. Free Radic Biol Med. 1987;3(5):313–6. doi: 10.1016/s0891-5849(87)80037-0 2826305

[43] Mercer AE, Maggs JL, Sun X-M, Cohen GM, Chadwick J, O’Neill PM, et al. Evidence for the involvement of carbon-centered radicals in the induction of apoptotic cell death by artemisinin compounds. J Biol Chem. 2007;282(13):9372–82. doi: 10.1074/jbc.M610375200 17227762

[44] Yan B, Ai Y, Sun Q, Ma Y, Cao Y, Wang J, et al. Membrane damage during ferroptosis is caused by oxidation of phospholipids catalyzed by the oxidoreductases POR and CYB5R1. Mol Cell. 2021;81(2):355–369.e10. doi: 10.1016/j.molcel.2020.11.024 33321093

[45] Do Q, Zhang R, Hooper G, Xu L. Differential contributions of distinct free radical peroxidation mechanisms to the induction of ferroptosis. JACS Au. 2023;3(4):1100–17. doi: 10.1021/jacsau.2c00681 37124288 PMC10131203

[46] Yi-Wen Z, Mei-Hua B, Xiao-Ya L, Yu C, Jing Y, Hong-Hao Z. Effects of oridonin on hepatic cytochrome P450 expression and activities in PXR-humanized mice. Biol Pharm Bull. 2018;41(5):707–12. doi: 10.1248/bpb.b17-00882 29709908

[47] Zhang Y-W, Zheng X-W, Liu Y-J, Fang L, Pan Z-F, Bao M-H, et al. Effect of oridonin on cytochrome P450 expression and activities in HepaRG Cell. Pharmacology. 2018;101(5–6):246–54. doi: 10.1159/000486600 29393278

[48] Morgan B, Lahav O. The effect of pH on the kinetics of spontaneous Fe(II) oxidation by O2 in aqueous solution--basic principles and a simple heuristic description. Chemosphere. 2007;68(11):2080–4. doi: 10.1016/j.chemosphere.2007.02.015 17368726

[49] Moore RE, Kim Y, Philpott CC. The mechanism of ferrichrome transport through Arn1p and its metabolism in *Saccharomyces cerevisiae*. Proc Natl Acad Sci U S A. 2003;100(10):5664–9. doi: 10.1073/pnas.1030323100 12721368 PMC156258

[50] Philpott CC, Protchenko O. Response to iron deprivation in *Saccharomyces cerevisiae*. Eukaryot Cell. 2008;7(1):20–7. doi: 10.1128/EC.00354-07 17993568 PMC2224162

[51] Phaniendra A, Jestadi DB, Periyasamy L. Free radicals: properties, sources, targets, and their implication in various diseases. Indian J Clin Biochem. 2015;30(1):11–26. doi: 10.1007/s12291-014-0446-0 25646037 PMC4310837

[52] Valko M, Leibfritz D, Moncol J, Cronin MTD, Mazur M, Telser J. Free radicals and antioxidants in normal physiological functions and human disease. Int J Biochem Cell Biol. 2007;39(1):44–84. doi: 10.1016/j.biocel.2006.07.001 16978905

[53] Murphy MP. How mitochondria produce reactive oxygen species. Biochem J. 2009;417(1):1–13. doi: 10.1042/BJ20081386 19061483 PMC2605959

[54] Redza-Dutordoir M, Averill-Bates DA. Activation of apoptosis signalling pathways by reactive oxygen species. Biochim Biophys Acta. 2016;1863(12):2977–92. doi: 10.1016/j.bbamcr.2016.09.012 27646922

[55] Carneiro BA, El-Deiry WS. Targeting apoptosis in cancer therapy. Nat Rev Clin Oncol. 2020;17(7):395–417. doi: 10.1038/s41571-020-0341-y 32203277 PMC8211386

[56] Bourez S, Van den Daelen C, Le Lay S, Poupaert J, Larondelle Y, Thomé J-P, et al. The dynamics of accumulation of PCBs in cultured adipocytes vary with the cell lipid content and the lipophilicity of the congener. Toxicol Lett. 2013;216(1):40–6. doi: 10.1016/j.toxlet.2012.09.027 23164672

[57] Mundy WR, Freudenrich TM, Crofton KM, DeVito MJ. Accumulation of PBDE-47 in primary cultures of rat neocortical cells. Toxicol Sci. 2004;82(1):164–9. doi: 10.1093/toxsci/kfh239 15282408

[58] Stadnicka-Michalak J, Tanneberger K, Schirmer K, Ashauer R. Measured and modeled toxicokinetics in cultured fish cells and application to in vitro-in vivo toxicity extrapolation. PLoS One. 2014;9(3):e92303. doi: 10.1371/journal.pone.0092303 24647349 PMC3960223

[59] Schreiber R, Altenburger R, Paschke A, Küster E. How to deal with lipophilic and volatile organic substances in microtiter plate assays. Environ Toxicol Chem. 2008;27(8):1676–82. doi: 10.1897/07-504.1 18318592

[60] Fischer FC, Cirpka OA, Goss K-U, Henneberger L, Escher BI. Application of experimental polystyrene partition constants and diffusion coefficients to predict the sorption of neutral organic chemicals to multiwell plates in in vivo and in vitro bioassays. Environ Sci Technol. 2018;52(22):13511–22. doi: 10.1021/acs.est.8b04246 30298728

[61] Faravelli T, Pinciroli M, Pisano F, Bozzano G, Dente M, Ranzi E. Thermal degradation of polystyrene. J Anal Appl Pyrolysis. 2001;60(1):103–21. doi: 10.1016/s0165-2370(00)00159-5

[62] Deltcheva E, Chylinski K, Sharma CM, Gonzales K, Chao Y, Pirzada ZA, et al. CRISPR RNA maturation by trans-encoded small RNA and host factor RNase III. Nature. 2011;471(7340):602–7. doi: 10.1038/nature09886 21455174 PMC3070239

[63] Hu M, Yuan X, Liu Y, Tang S, Miao J, Zhou Q, et al. IL-1β-induced NF-κB activation down-regulates miR-506 expression to promotes osteosarcoma cell growth through JAG1. Biomed Pharmacother. 2017;95:1147–55. doi: 10.1016/j.biopha.2017.08.120 28926924

[64] Jinek M, East A, Cheng A, Lin S, Ma E, Doudna J. RNA-programmed genome editing in human cells. Elife. 2013;2:e00471. doi: 10.7554/eLife.00471 23386978 PMC3557905

[65] Kaelin WG Jr. The concept of synthetic lethality in the context of anticancer therapy. Nat Rev Cancer. 2005;5(9):689–98. doi: 10.1038/nrc1691 16110319

[66] Zhu J, Pan S, Chai H, Zhao P, Feng Y, Cheng Z, et al. Microfluidic impedance cytometry enabled one-step sample preparation for efficient single-cell mass spectrometry. Small. 2024;20(26):e2310700. doi: 10.1002/smll.202310700 38483007

[67] Tong G, Peng T, Chen Y, Sha L, Dai H, Xiang Y, et al. Effects of GLP-1 receptor agonists on biological behavior of colorectal cancer cells by regulating PI3K/AKT/mTOR signaling pathway. Front Pharmacol. 2022;13:901559. doi: 10.3389/fphar.2022.901559 36034798 PMC9399678

[68] Fujii H, Kakinuma K. Direct measurement of superoxide anion produced in biological systems by ESR spectrometry: a pH-jump method. J Biochem. 1990;108(6):983–7. doi: 10.1093/oxfordjournals.jbchem.a123325 1965191

